# **Diethylstilbestrol (DES)-Stimulated Hormonal Toxicity is Mediated by ER**α **Alteration of Target Gene Methylation Patterns and Epigenetic Modifiers (*DNMT3A*, *MBD2*, and *HDAC2*) in the Mouse Seminal Vesicle**

**DOI:** 10.1289/ehp.1307351

**Published:** 2013-12-06

**Authors:** Yin Li, Katherine J. Hamilton, Anne Y. Lai, Katherine A. Burns, Leping Li, Paul A. Wade, Kenneth S. Korach

**Affiliations:** 1 Laboratory of Reproductive and Developmental Toxicology,; 2 Laboratory of Molecular Carcinogenesis, and; 3 Biostatistics Branch, National Institute of Environmental Health Sciences, National Institutes of Health, Department of Health and Human Services, Research Triangle Park, North Carolina, USA

## Abstract

Background: Diethylstilbestrol (DES) is a synthetic estrogen associated with adverse effects on reproductive organs. DES-induced toxicity of the mouse seminal vesicle (SV) is mediated by estrogen receptor α (ERα), which alters expression of seminal vesicle secretory protein IV (*Svs4*) and lactoferrin (*Ltf*) genes.

Objectives: We examined a role for nuclear receptor activity in association with DNA methylation and altered gene expression.

Methods: We used the neonatal DES exposure mouse model to examine DNA methylation patterns via bisulfite conversion sequencing in SVs of wild-type (WT) and ERα-knockout (αERKO) mice.

Results: The DNA methylation status at four specific CpGs (–160, –237, –306, and –367) in the *Svs4* gene promoter changed during mouse development from methylated to unmethylated, and DES prevented this change at 10 weeks of age in WT SV. At two specific CpGs (–449 and –459) of the *Ltf* gene promoter, DES altered the methylation status from methylated to unmethylated. Alterations in DNA methylation of *Svs4* and *Ltf* were not observed in αERKO SVs, suggesting that changes of methylation status at these CpGs are ERα dependent. The methylation status was associated with the level of gene expression. In addition, gene expression of three epigenetic modifiers—*DNMT3A, MBD2*, and *HDAC2*—increased in the SV of DES-exposed WT mice.

Conclusion: DES-induced hormonal toxicity resulted from altered gene expression of *Svs4* and *Ltf* associated with changes in DNA methylation that were mediated by ERα. Alterations in gene expression of *DNMT3A, MBD2*, and *HDAC2* in DES-exposed male mice may be involved in mediating the changes in methylation status in the SV.

Citation: Li Y, Hamilton KJ, Lai AY, Burns KA, Li L, Wade PA, Korach KS. 2014. Diethylstilbestrol (DES)-stimulated hormonal toxicity is mediated by ERα alteration of target gene methylation patterns and epigenetic modifiers (*DNMT3A*, *MBD2*, and *HDAC2*) in the mouse seminal vesicle. Environ Health Perspect 122:262–268; http://dx.doi.org/10.1289/ehp.1307351

## Introduction

Endocrine-disrupting chemicals (EDCs) are substances in the environment, food sources, and manufactured products that can interfere with the normal functioning of the body’s endocrine system (Diamanti-Kandarakis et al. 2009). EDCs include synthetized or natural hormones, pharmaceuticals, pesticides, and plasticizers that influence activity of estrogen receptors (ERs) (Henley et al. 2009). Diethylstilbestrol (DES) was the first orally active synthetic estrogen, and its use was intended to facilitate placental steroidogenesis and reduce the risk of spontaneous abortion or preterm parturition in pregnant women (Marselos and Tomatis 1992a, 1992b). In clinical studies in 1971, DES was found to cause a rare vaginal tumor in young women exposed to this drug *in utero* (Greenwald et al. 1971; Herbst et al. 1971). Almost immediately after the reports were published, the U.S. Food and Drug Administration blocked the use of DES for pregnancy support (Herbst 2000).

A mouse model of neonatal DES exposure has been widely used to study the possible effects of DES on the reproductive organs (McLachlan 1977; McLachlan and Dixon 1977). This model system has also been used to help elucidate the mechanism(s) of hormonal carcinogenicity (McClain et al. 2001). Studies have indicated that female mice treated neonatally with DES develop a high incidence of uterine adenocarcinoma (Newbold et al. 1990). Similarly treated male mice develop testicular cancer and abnormalities of the prostate and seminal vesicles (SVs) (McLachlan 1977; McLachlan and Dixon 1977). Prins and colleagues reported that neonatal estrogen (E2) exposure induced lobe-specific alterations in the adult rat prostate, including a permanent decrease in androgen receptor (AR) levels (Prins 1992; Prins et al. 1993). In a study using the neonatal DES model, Prins and Bremner (2004) reported an abnormal morphology of the penis in male rats associated with changes in the protein levels of ERα, but not AR. In addition, neonatal DES exposure has been reported to significantly decrease the level of ERα protein in the anterior prostate but increase its level in the SV of male mice (Turner et al. 1989).

The biological effects of E2 and some EDCs are mediated through the ERs (ERα and ERβ), which are members of a large superfamily of nuclear receptors. These receptors act as ligand-inducible transcription factors (Hall and McDonnell 2005). The classical mechanism of ER action is characterized by ER directly binding to estrogen response elements (EREs) of target genes. The nonclassical mechanism is the “tethered” mechanism, in which ERs regulate the expression of a large number of E2-responsive genes through interaction with other transcription factors, such as c-Jun, c-Fos, or Stat5 (Björnström and Sjöberg 2005). EDCs regulate many target genes through the ER, similar to the regulation by E2 (Moggs et al. 2004).

Seminal vesicle secretory protein IV (SVS4) is an androgen-dependent protein (Chen et al. 1987). The expression of the *Svs4* gene is dependent on the presence of testosterone in the rat SV (Higgins et al. 1976, 1981). Lactoferrin (or lactotransferrin; *Ltf* ) is a female-specific gene and serves as an appropriate marker of estrogenic action because of its high level of RNA and protein expression in E2-stimulated uteri compared with other tissues (Pentecost and Teng 1987). Prenatal DES exposure studies have shown that the expression levels of the *Ltf* gene are induced in the SV of DES-treated mice (Newbold et al. 1989).

Our research group has used the ER-knockout (ERKO) mouse to study ER-dependent pathways involved in mediating the effects of neonatal DES exposure in the reproductive tract tissues (Couse et al. 2001; Couse and Korach 1999). Results of those studies demonstrated that ERα plays a critical role in mediating the toxicological effects of neonatal DES exposure in female and male reproductive tracts. In the prostate, E2 imprinting of the developing prostate gland was mediated through stromal ERα (Prins et al. 2001). In 4-month-old mice, neonatal DES exposure resulted in decreased SV weight in wild-type (WT) mice but not in αERKO mice (Couse and Korach 2004; Prins et al. 2001). Recently, our laboratory reported that DES-induced SV toxicity and feminization were primarily mediated through ERα in adult mice (Walker et al. 2012).

DNA methylation is a well-characterized epigenetic modification and is important for gene regulation, transcriptional silencing, development, and tumorigenesis (Esteller 2008; Feinberg and Tycko 2004; Jones and Baylin 2007; Wu and Zhang 2010). In mammalian cells, DNA methylation occurs at the 5´ position of the cytosine ring within CpG dinucleotides via addition of a methyl group to create a 5´-methylcytosine. The methylation at the 5´-methylcytosine is catalyzed by DNA methyltransferases (DNMTs), including *DNMT1*, *DNMT3A*, and *DNMT3B* (Bestor 2000; Chen and Li 2004). The DNA methylation pattern is believed to be “read” by a conserved family protein, the methyl CpG binding domain (MBD) family (Jaenisch and Bird 2003; Wade 2001). The MeCP2, MBD2, and MBD3 proteins belong to the MBD family and represent an important class of chromosomal proteins, which associate with protein partners that play active roles in transcriptional repression and/or heterochromatin formation (Wade 2005). The second well-known epigenetic mechanism is histone modification, which is critical for regulating chromatin structure and function (Jenuwein and Allis 2001; Luger et al. 1997). Histone deacetylases (HDACs) 1 and 2 are highly conserved enzymes that help regulate chromatin structure as the core catalytic components of corepressor complexes (Brunmeir et al. 2009). To date, studies indicate that these epigenetic markers play an important role in transcriptional programs during development. However, the correlation between DNA methylation and gene expression, as well as the involvement of these epigenetic markers, in response to EDC exposure are still poorly understood.

In the present study, we used a neonatal mouse model of DES exposure to examine the changes of DNA methylation patterns in the altered androgen-dependent gene *Svs4* and in the estrogen-dependent gene *Ltf*, as well as the correlation of their methylation status with gene expression. Furthermore, we evaluated the role of ERα in the DNA methylation process and the role of altered gene expression of epigenetic markers in the seminal vesicle of male mice.

## Materials and Methods

*Chemicals*. Diethylstilbestrol (DES; CAS no. 56-53-1) was purchased from Sigma-Aldrich (St. Louis, MO).

*Animals and neonatal treatment*. All animal studies were conducted in accordance with the *Guide for the Care and Use of Laboratory Animals* (Institute for Laboratory Animal Research 2011) and approved by the National Institute of Environmental Health Sciences Animal Care and Use Committee. Animals were treated humanely and with regard for alleviation of suffering. Mice were housed under constant environmental conditions (22 ± 1°C; relative humidity, 40–60%; 12:12-hr light:dark cycle). Mice received autoclaved feed (NIH31 pelleted chow; Zeigler Brothers, Gardners, PA) and reverse-osmosis deionized water *ad libitum*. They were housed in polycarbonate caging with hardwood bedding (SaniChip; PJ Murphy Forest Products, Montville, NJ) with autoclaved environmental enrichment (Nestlets; Ancare, Bellmore, NY). For WT mice, 8- to 12-week-old pregnant C57BL/6 females (*n* = 30) were obtained from Charles River Laboratories (Wilmington, MA). ERα-null mice (αERKO) were generated by breeding C57BL/6 heterozygous (ERα^+/–^) animals as described previously (Couse et al. 2003). On the day of birth (considered day 1), male pups from multiple litters were pooled and randomly distributed among 8- to 12-week-old CD-1 foster mothers with eight pups per dam. For neonatal treatment, pups were treated each morning of days 1–5 by subcutaneous injection with either DES dissolved in corn oil (2 μg/pup/day; 0.02 cc) DES group) or an equal volume of corn oil (vehicle group). Mice (WT and αERKO) were weaned and genotyped on day 21. After weaning, mice were housed two to four per cage by treatment group. The genotyping was performed by polymerase chain reaction (PCR) on DNA extracted from tail biopsy using previously described methods (Couse et al. 2003).

**Figure 1:**
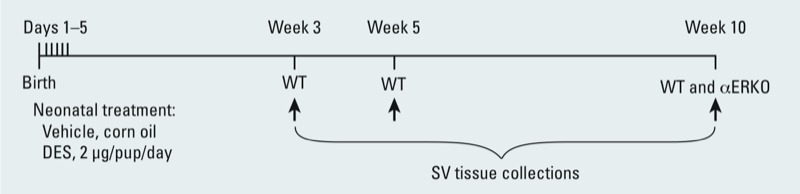
Timeline for neonatal treatment [DES (2 μg/day) or corn oil vehicle (2 μg/day)] and tissue collection.

SVs were collected at weeks 3, 5, and 10 from WT mice (vehicle and DES groups) and only at week 10 from both treatment groups of αERKO mice (Figure 1). On the day of tissue collection, mice were euthanized by CO_2_ inhalation and SVs were removed immediately. A portion of each SV was fixed in Bouins solution for immunohistochemistry. The remaining SV tissues were snap frozen and stored at –80°C until use.

*LF immunohistochemistry.* For immunohistochemistry, SVs were fixed in Bouin’s solution, processed through graded ethanol, embedded in paraffin, and sectioned at 7 μm. See Supplemental Material, p. 4, for additional details. After deparaffinization, slides were immunostained using anti-LF antibody (sc-14434; Santa Cruz Biotechnology, Santa Cruz, CA) and the AEC (aminoethyl carbazole) kit (Zymed Laboratory, South San Francisco, CA) according to the manufacturer’s instructions. Tissues were then counterstained with hematoxylin (Sigma-Aldrich).

*RNA extraction and real-time PCR*. Total RNA samples were extracted from frozen SV tissue from individual mice using the RNeasy Mini Kit (QIAGEN, Valencia, CA) according to the manufacturer’s protocol. First-strand cDNA synthesis was performed using Superscript Reverse Transcriptase (Invitrogen, Carlsbad, CA) according to the manufacturer’s protocol. We measured mRNA levels *Svs4*, *Ltf*, *Pgr* (progesterone receptor), *Stat3*, *Stat5a*, *DNMT1*, *DNMT3A*, *DNMT3B*, *MeCP2*, *MBD2*, *MBD3*, *HDAC1*, and *HDAC2* using SYBR green assays (Applied Biosystems, Foster City, CA). The sequences of primers used in real-time PCR are provided in Supplemental Material, Table S1. Cycle threshold (Ct) values were obtained using the ABI PRISM 7900 Sequence Detection System and analysis software (Applied Biosystems). The experiments were repeated three times, and results are presented as the fold increase (± SE) calculated relative to the vehicle WT group at week 5.

*Identification of potential ERE sequences and CpGs*. We downloaded the genomic sequence of the gene promoters (*Svs4*, *Ltf*, and *Pgr*) from the UCSC Genome Browser (http://genome.ucsc.edu; build mm10). A putative ERE sequence with the position weight matrix (PWM) constructed from 48 experimentally identified EREs (15 bp in length) was scanned using GADEM software (Jin et al. 2004; Li 2009). CpGs were identified using EpiDesigner software (http://www.epidesigner.com/).

*DNA extraction and bisulfite conversion–sequencing PCR*. Genomic DNA (400–500 ng) was extracted from frozen SV tissue of individual mice using a Tissue Blood Kit (QIAGEN) according to the manufacturer’s protocol. Bisulfite conversion–sequencing PCR primers (see Supplemental Material, Table S2) were designed using EpiDesigner. Bisulfite conversion–sequencing PCR was performed using the EZ DNA Methylation-Gold Kit (Zymo Research, Irvine, CA) following the manufacturer’s instructions. The PCR products were resolved on a 2% agarose gel and purified using a QIAquick Gel Extraction Kit (QIAGEN).

*Cloning and sequencing bisulfite-treated DNA*. Purified PCR product from individual mice was subcloned into the pCR-TOPO-XL vector using the TOPO XL PCR Cloning Kit (Invitrogen) following the manufacturer’s instructions. Six or more clones were selected and sequenced for each sample. We performed the sequencing analysis of bisulfite-treated DNA using CpGviewer software (http://dna.leeds.ac.uk/cpgviewer/) (Carr et al. 2007). Data presented represent three individual mice per treatment group.

*Statistical analysis*. One-way analysis of variance (ANOVA) with Dunnett’s multiple comparison test (*p* < 0.05 and *p* < 0.01) and two-way ANOVA with Bonferroni posttest (*p* < 0.001) were performed using GraphPad Prism, version 6.00 (GraphPad Software, San Diego, CA, USA).

## Results

**Figure 2:**
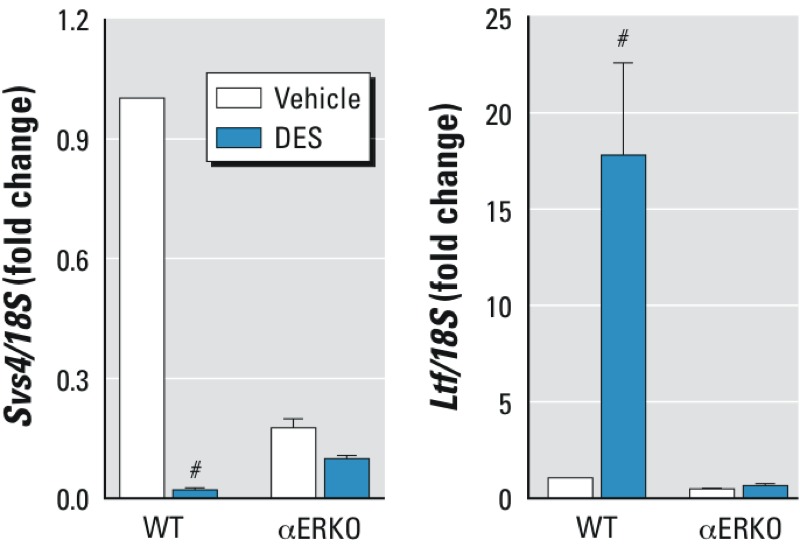
Effect of neonatal DES exposure on *Svs4* (*A*) or *Ltf* (*B*) gene expression in SVs of mice 10 weeks after treatment with vehicle or DES. Total RNA samples were extracted from SV tissues of three individual WT or αERKO mice per treatment group, and mRNA levels were quantified by real time-PCR. Data shown represent mean fold change (± SE) relative to SVs from WT vehicle-treated mice at week 5. ^#^*p* < 0.001 by two-way ANOVA with Bonferroni posttest.

*DES-altered levels of* Svs4 *and* Ltf *gene expression are ER*α *dependent.* To verify DES alteration of androgen- or estrogen-dependent genes in SV tissues, we examined *Svs4* (a male-specific gene) and *Ltf* (a female-specific gene) in adult WT and αERKO male mice treated neonatally with either DES or vehicle. The *Svs4* gene is highly expressed in the WT SV. In SVs collected 10 weeks after neonatal treatment, the expression level of *Svs4* in the DES group was < 10% that in the vehicle group (Figure 2A). *Svs4* expression was much lower in αERKO vehicle group than in WT vehicle group. In αERKO mice, we observed no significant change between DES- and vehicle-treated mice (Figure 2A). In addition, at week 5, expression of *Svs4* was lower in both WT vehicle and DES groups than in WT vehicle group at week 10 (data not shown).

At week 10, we found minimal *Ltf* gene expression in SVs from both WT and αERKO mice treated with vehicle (Figure 2B). Interestingly, in DES-treated animals, we found high levels of *Ltf* gene expression in WT but not αERKO SVs. In addition, when we used immunohistochemical staining to examine the levels of LF protein, we found strong staining in SVs from DES-treated WT but not DES-treated αERKO mice (see Supplemental Material, Figure S1). These data show that ERα mediates DES-induced alterations of gene expression in the SV of adult male mice.

**Figure 3:**
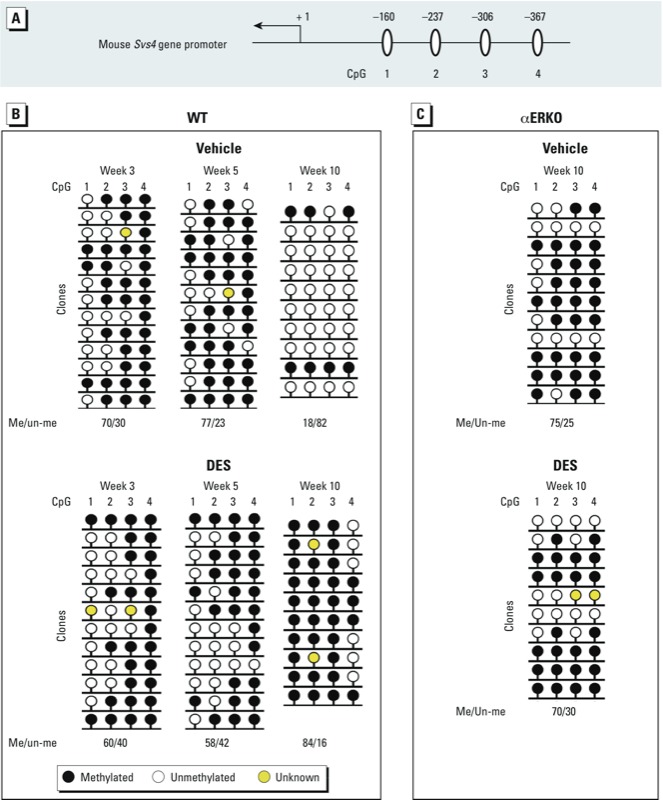
The methylation status of *Svs4* changes during development after neonatal treatment with vehicle or DES. (*A*) Diagram depicting four CpGs in the mouse *Svs4* gene. (*B*,*C*) DNA methylation status of the *Svs4* gene in SVs from WT mice at weeks 3, 5, and 10 (*B*) and from αERKO mice at week 10 (*C*). Genomic DNA was extracted from SV tissues of individual mice, the region containing the four CpGs was amplified by PCR from bisulfite-treated genomic DNA, and the PCR product was subcloned into the pCR-TOPO-XL vector. The sequencing analysis was performed using CpGviewer. Each line of circles indicates an individual clone sequenced in the analysis. Data shown represent the results from three individual mice per group. The percentages of methylated/unmethylated (Me/un-me) CpGs represent the results from all four CpGs.

*Methylation status of four specific CpGs in the* Svs4 *gene promoter: effect of DES and ER*α *on change from methylated to unmethylated during development.* Using data from UCSC Genome Browser, we found four CpGs (–160, –237, –306, and –367) located in the *Svs4* gene promoter close to the transcription start site (Figure 3A). To determine whether DNA methylation correlates with *Svs4* transcription, we used bisulfite sequencing to examine the methylation status of these four CpGs in WT SVs at weeks 3, 5, and 10. In vehicle group, > 70% of these CpGs were methylated at weeks 3 and 5, but only 18% of the same CpGs were methylated at week 10 (Figure 3B, top). However, in the DES-treated WT mice, about 60% of the CpGs were methylated at week 3 or 5 and 84% were methylated at week 10 (Figure 3B, bottom). The maintenance of methylation at these CpGs is consistent with down-regulation of the *Svs4* gene in the DES WT group at week 10 (Figure 2A).

To examine the effects of ERα on the DNA methylation status of the *Svs4* gene promoter, we performed bisulfite conversion sequencing PCR with SV tissue collected from αERKO mice at week 10. In both vehicle and DES αERKO groups, > 70% of the four CpGs were methylated (Figure 3C). These results suggest that the lack of ERα and neonatal DES treatment each blocked the normal developmental alterations in the DNA methylation status of these four specific CpGs (–160, –237, –306, and –367) of the *Svs4* gene promoter. The observed alterations of DNA methylation correlate with gene expression.

**Figure 4:**
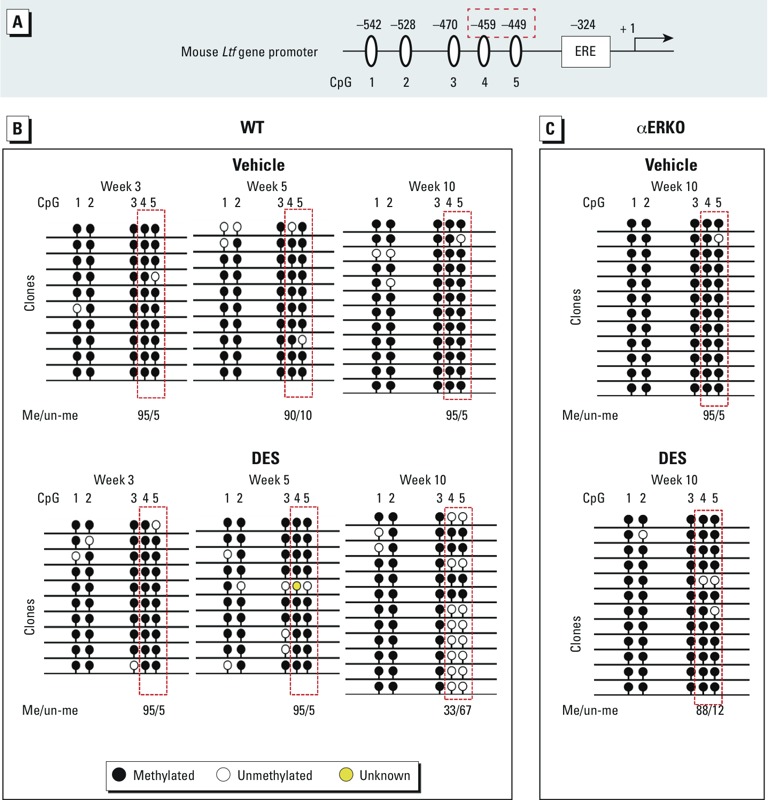
The methylation status of *Ltf* changes during development after neonatal treatment with vehicle or DES. (*A*) Diagram depicting five CpGs in the mouse *Ltf* gene promoter. (*B*,*C*) DNA methylation status of the *Ltf* gene in SVs from WT mice at weeks 3, 5, and 10 (*B*) and from αERKO mice at week 10 (*C*). Genomic DNA was extracted from SV tissues of individual mice, the region containing the five CpGs was amplified by PCR from bisulfite-treated genomic DNA, and the PCR product was subcloned into the pCR-TOPO‑XL vector. The sequencing analysis was performed using CpGviewer. Each line of circles indicates an individual clone sequenced in the analysis. Data shown represent the results from three individual mice per group. The percentages of methylated/unmethylated (Me/un-me) CpGs represent the results from two CpGs (–449 and –459).

*Methylation status of two specific CpGs in the* Ltf *gene promoter: effect of neonatal DES treatment on changes from methylated to unmethylated.* In the *Ltf* gene promoter, we found five CpGs (–449, –459, –470, –528, and –542) located close to a well-characterized ERE site (–324) (Li et al. 1997; Liu and Teng 1992) and the transcription start site (Figure 4A). To determine whether the alteration of DNA methylation directly regulates *Ltf* gene transcription, we examined the methylation status of these five CpGs in the *Ltf* gene promoter in the WT SV collected at weeks 3, 5, and 10 after neonatal DES treatment. In the vehicle group, > 90% of these CpGs were methylated, and the methylation pattern did not change during development, from weeks 3 to 10 (Figure 4B, top). In SVs from the DES-treated WT mice, there were no changes in the methylation status at week 3 or week 5; however, at week 10, two specific CpGs (–449 and –459) changed from methylated to unmethylated (Figure 4B, bottom). Loss of methylation at these CpGs is consistent with the up-regulation of *Ltf* gene expression in the DES group at week 10 (Figure 2B).

We also examined the methylation status of the same five CpGs in the *Ltf* gene promoter in SVs from αERKO mice at week 10. In DES-treated mice, we found no change in the methylation status of two specific CpGs (–449 and –459) (Figure 4C). These data suggest that DES altered the methylation status of these two CpGs in the *Ltf* gene promoter from methylated to predominantly unmethylated, and that the absence of ERα blocked this change.

*Differential effects of neonatal DES treatment on the expression levels of the epigenetic modifiers* DNMT3A, MBD2, *and* HDAC2. To examine whether the altered methylation patterns in SVs from mice treated neonatally with DES might be affected by the expression level of the epigenetic modifiers, we used real-time PCR to investigate the RNA levels of DNA methyltransferases, including DNMTs (*DNMT1*, *DNMT3A*, and *DNMT3B*) and MBDs (*MeCP2*, *MBD2,* and *MBD3*), as well as two histone modifiers (*HDAC1* and *HDAC2*). We examined the expression levels of these genes in SVs collected from WT mice at week 5 or week 10 after neonatal exposure to vehicle or DES.

**Figure 5:**
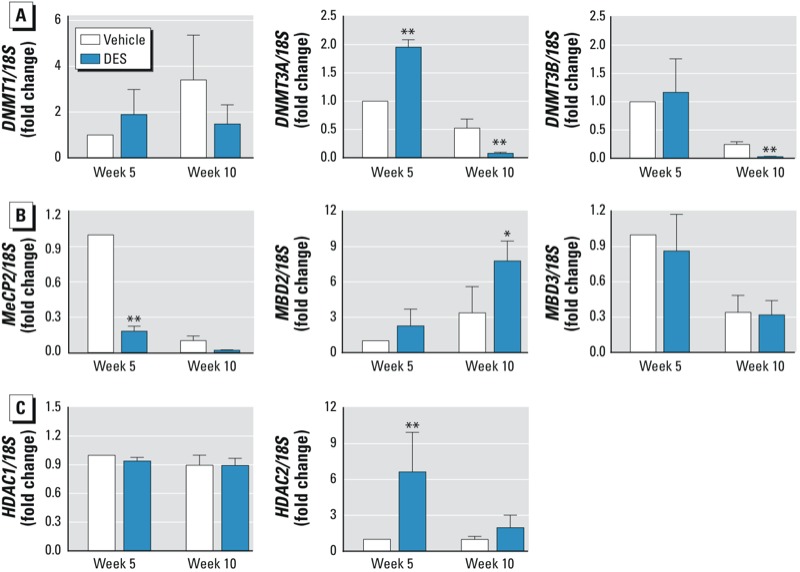
Gene expression levels of epigenetic markers DNMTs (*A*), MBDs (*B*), and HDACs (*C*) in WT SVs from mice treated neonatally with vehicle or DES. Total RNA samples were extracted from SV tissues of three individual WT or αERKO mice at week 5 or week 10 after neonatal treatment. Gene expression levels were quantified by real time-PCR. Data shown represent mean fold change (± SE) relative to SVs from WT vehicle-treated mice at week 5. *p < 0.05, and **p < 0.01 by one-way ANOVA with Dunnett’s multiple comparison test.

At week 5, the expression level of *DNMT3A* was significantly increased in the DES group compared with the vehicle group, but at week 10, *DNMT3A* was lower in the DES group than in the vehicle group (Figure 5A). However, the gene expression of *DNMT1* and *DNMT3B* was not significantly different between DES and vehicle groups in either week (Figure 5A). At week 5, the expression level of *MeCP2* was much higher in the vehicle group, but this level was significantly lower in the DES group (Figure 5B). However, we observed no significant difference in *MeCP2* gene expression between the vehicle and DES groups at week 10. *MBD2* expression at week 10 was significantly elevated in the DES group relative to the vehicle group at week 10. In addition, DES did not affect the expression of *MBD3* relative to vehicle at week 5 or week 10 (Figure 5B).

Expression of the histone modification marker *HDAC2* was significantly increased in SVs from the DES group compared with the vehicle group at week 5 but not at week 10. In contrast, expression of *HDAC1* was not different in the DES group at either week 5 or week 10 (Figure 5C). These findings suggest that alterations in gene expression of these epigenetic modifiers are correlated with changes in methylation status seen with neonatal DES treatment.

## Discussion

*Correlation between altered DNA methylation and the expression levels of specific genes in SVs of adult male mice neonatally treated with DES*. There is increasing interest in the effect of EDCs on human health (Henley et al. 2009). One of the earliest recognized EDCs, DES is still being used to study the possible effects of EDCs on reproductive organs. Previous studies in our laboratory observed alterations in male- and female-specific genes in adult male mice treated neonatally with DES (Couse and Korach 2004; Walker et al. 2012). In the present study, we investigated the effect of neonatal DES treatment on DNA methylation patterns of *Svs4* (male-specific) and *Ltf* (female-specific) genes during development in SVs from WT and αERKO mice. One of the most significant findings of our study is a correlation between DNA methylation patterns and the levels of *Svs4* and *Ltf* gene expression in an ERα-dependent manner in DES-treated mice.

Previous studies have shown that the decrease in *Svs4* expression is not due to a significant change in the level of *AR* gene expression in the SVs of adult male mice neonatally treated with DES (Turner et al. 1989; Walker et al. 2012). SVS IV protein is found in the SVs of mice and rats and is regulated by androgen (Chen et al. 1987). In the present study, we used data from UCSC Genome Browser to analyze the Svs4 gene promoter. We found a putative Stat5a/5b binding site upstream (–132/–146 bp) and an Sp1 site downstream (+118/+128 bp) (see Supplemental Material, Figure S2). We found a predicted ERE site within the 2 kb of the transcription start site (+/–1 kb). However, the 4 bases of the palindrome differ from the 10 base consensus ERE (GGTCAnnnTGACC) (see Supplemental Material, Figure S2). Couse and Korach (2004) found that SV weight was significantly lower in DES-treated WT adult male mice but not in DES-treated αERKO mice. These data suggest that this effect is ERα dependent and might act through the nonclassical (tethered) mechanism.

In the SV tissues from rat, a methylation-sensitive restriction assay showed that seven potential methylation sites were largely methylated (Kandala et al. 1985). In the present study, we found a normal developmental change in DNA methylation status at four specific CpGs (–160, –237, –306, and –367) of the *Svs4* gene promoter in WT SVs between week 3 and week 10. Furthermore, DES exposure and the absence of ERα both blocked the normal developmental demethylation of the *Svs4* gene; these changes in DNA methylation correlate with the gene expression findings in this study. This is the first report addressing the correlation between DNA methylation and expression of the *Svs4* gene in a mouse model, as well as a role for ERα in this process. Our results help to explain the relationships of epigenetic mechanisms and gene regulation.

The *Ltf* gene, a well-known female-specific ER target gene, is up-regulated by E2 in the female reproductive tract (Teng 2002). In female mice, the early appearance of LF protein expression suggests that it may play an important role in the hormonal regulation of growth and differentiation of developing uterine tissues (Newbold et al. 1997). In male mice, there is normally no *Ltf* gene expression, nor is there a potential role for this gene in SV tissues; however, *Ltf* is highly expressed after neonatal/prenatal DES exposure (Couse and Korach 2004; Newbold et al. 1989). E2 increases *Ltf* expression through a well-characterized ERE (–324) located upstream from the *Ltf* promoter transcription start site (Liu and Teng 1992; Liu et al. 1993). Using chromatin immunoprecipitation (ChIP) quantitative PCR (Hewitt et al. 2010), we confirmed that ERα is bound to this ERE site (–324) in the *Ltf* gene promoter in SVs from 10-week-old WT mice with or without neonatal DES exposure. However, DES enhances the enrichment of ERα binding (data not shown). In addition, we found three predicted EREs and an Sp1 site in the region 1 kb upstream of the *Ltf* gene promoter (see Supplemental Material, Figure S3). Importantly, in SVs from male mice neonatally treated with DES, we found two specific CpGs (–449 and –459) upstream of the *Ltf* gene promoter that have altered DNA methylation status (from methylated to unmethylated). In a methylation analysis of the *Ltf* gene promoter in the CD-1 mouse uterus, Li et al. (1997) observed that prenatal DES exposure altered only one CpG site (CpG site –464, corresponding with CpG site –459 in our study), which changed from methylated to unmethylated; this finding suggsts that the effects of DES on DNA methylation are tissue specific. Also in that study (Li et al. 1997), DNA methylation status in the *Ltf* promoter of the uterus of 3-week-old CD-1 mice changed from methylated to unmethylated at three specific CpGs (–475, –533, and –547), corresponding with CpG sites –470, –528, and –542 in the present study. At week 3 in the present study, we observed no developmental changes at these three CpGs (–470, –528, and –542) in SV tissues from C57BL/6 male mice. These findings suggest that there are sex and/or strain differences in the DNA methylation patterns of the *Ltf* gene in the mouse reproductive organs.

Next, we examined the gene expression level of *Pgr*, a well-known ER target gene, in SVs. The gene profile showed differences in expression of the *Pgr* gene in vehicle-treated WT and αERKO mice due to the lack of ERα, but there were no significant changes of *Pgr* expression in the SVs of DES-treated WT and αERKO mice (see Supplemental Material, Figure S4A). Using EpiDesigner software, we found a high CpG content, with 20 CpGs located in the introns of the *Pgr* gene between +661 and +886. When examining the DNA methylation patterns, we found that almost 100% of these CpGs were unmethylated in SVs of both vehicle and DES groups at week 10 (see Supplemental Material, Figure S4B), suggesting that the DNA methylation patterns of the *Pgr* gene correlate with its gene expression. There might be other CpGs of the *Pgr* gene involved in reduction of gene expression in αERKO SVs compared with WT SVs; however, in the αERKO mouse, it is most likely that the lack of ERα and the hormone responsiveness in the SV explain the lower expression of *Pgr*.

The growth-hormone-signaling–activated transcription factors *Stat3* and *Stat5a* regulate estrogen signaling (Yamamoto et al. 2000; Hewitt et al. 2010). In the present study, we found that the expression levels of *Stat3* and *Stat5a* in SVs from DES-treated WT mice were significantly increased at week 5 but not at week 10 (see Supplemental Material, Figure S5). Furthermore, when we focused our efforts on ERα, we reconfirmed that there were no changes in *AR* gene expression in SVs from DES-treated mice compared with those treated with vehicle (data not shown), suggesting that alterations of *Pgr* in SVs of DES-treated males occurred through another nuclear receptor. In addition, the level of serum testosterone is lower in adult αERKO males compared with WT males, and this may regulate the levels of *Svs4* and *Ltf* gene expression (Walker et al. 2012).

*Neonatal DES treatment alters the expression levels of epigenetic modifiers in the SVs of male mice*. There are two main epigenetic mechanisms/modifications: DNA methylation and histone modification (Gabory et al. 2011). The enzymatic machinery for DNA methylation is composed mainly of three DNMTs: *DNMT1*, *DNMT3A*, and *DNMT3B* (Bestor 1988). In recent years, the levels of these enzymes have been measured in reproductive organs and used as DNA methylation markers after exposure to EDCs, such as DES and bisphenol A (BPA) (Bromer et al. 2010; Sato et al. 2006, 2009). In mice neonatally treated with DES, expression levels of *DNMT1*, *DNMT3A*, and *DNMT3B* in the uterus and epididymis change dynamically (Sato et al. 2006, 2009). In the present study we found that only *DNMT3A* expression increased in the SVs mice neonatally exposed to DES (at the 5-week time point), suggesting that *DNMT3A* is involved in epigenetic programming at different periods of development. Interestingly, expression of *DNMT3A* and *DNMT3B* was much lower in the DES group than in the vehicle group at week 10, indicating that these epigenetic modifiers change dynamically during DES exposure.

The MBD family of proteins (e.g., *MeCP2*, *MBD2*, and *MBD3*) play an important role in transcriptional repression (Bird et al. 1999; Hendrich and Tweedie 2003; Wade 2001). We found a significant increase in *MBD2* expression in DES-treated mice at week 10 in the present study. This finding is in agreement with the study of Tang et al. (2012), which found that *MBD2* expression increased after neonatal E2/BPA exposure in the rat prostate gland.

The histone modification markers (*HDACs*) are evolutionarily conserved enzymes that remove acetyl modifications from histones and play a central role in epigenetic gene silencing (Hayakawa and Nakayama 2011). Dovey et al. (2010) found that *HDAC1* controlled embryonic stem cell differentiation, but they observed no effect of *HDAC2.* In DES-treated mice in the present study, we observed that *HDAC2* expression was increased significantly in SVs at week 5. These data indicate that the involvement of these histone modifiers in epigenetic programming could be cell- and tissue-type specific. The observed changes in these proteins suggest that the effects of DES on DNA methylation of target genes may be more widespread, and a global analysis needs to be performed in future studies.

## Conclusions

In the present study we found an association between DNA methylation and gene expression for the *Svs4* and *Ltf* genes. A working model of this study is shown in Supplemental Material, Figure S6. Four specific CpGs (–160, –237, –306, and –367) in the *Svs4* gene changed from methylated to unmethylated during development; however, methylation changes at these CpGs were not observed in mice neonatally treated with DES. Normal methylation changes in the *Svs4* gene were not seen in αERKO mice, suggesting that ERα may play an active role in the methylation changes. In WT mice, DES altered the DNA methylation status from methylated to unmethylated at two specific CpGs (–449 and –459) in the *Ltf* gene promoter. In addition, DES treatment appeared to significantly regulate the expression levels of the epigenetic modifiers *DNMT3A*, *MBD2*, and *HDAC2*. Taken together, these results are consistent with the hypothesis that DES-induced toxicity is mediated by ERα alteration of target gene methylation patterns and through changes in gene expression of three epigenetic modifiers in the SV of adult male mice neonatally treated with DES.

## Supplemental Material

(1.3 MB) PDF

## References

[r1] Bestor TH (1988). Cloning of a mammalian DNA methyltransferase.. Gene.

[r2] Bestor TH (2000). The DNA methyltransferases of mammals.. Hum Mol Genet.

[r3] Bird AP, Wolffe AP (1999). Methylation-induced repression—belts, braces, and chromatin.. Cell.

[r4] Björnström L, Sjöberg M (2005). Mechanisms of estrogen receptor signaling: convergence of genomic and nongenomic actions on target genes.. Mol Endocrinol.

[r5] Bromer JG, Zhou Y, Taylor MB, Doherty L, Taylor HS (2010). Bisphenol-A exposure *in utero* leads to epigenetic alterations in the developmental programming of uterine estrogen response.. FASEB J.

[r6] Brunmeir R, Lagger S, Seiser C (2009). Histone deacetylase HDAC1/HDAC2-controlled embryonic development and cell differentiation.. Int J Dev Biol.

[r7] Carr IM Valleley EM Cordery SF Markham AF Bonthron DT 2007 Sequence analysis and editing for bisulphite genomic sequencing projects. Nucleic Acids Res 35 10 e79; 10.1093/nar/gkm330 17517768 PMC1904293

[r8] Chen T, Li E (2004). Structure and function of eukaryotic DNA methyltransferases.. Curr Top Dev Biol.

[r9] Chen YH, Pentecost BT, McLachlan JA, Teng CT (1987). The androgen-dependent mouse seminal vesicle secretory protein IV: characterization and complementary deoxyribonucleic acid cloning.. Mol Endocrinol.

[r10] Couse JF, Dixon D, Yates M, Moore AB, Ma L, Maas R (2001). Estrogen receptor-α knockout mice exhibit resistance to the developmental effects of neonatal diethylstilbestrol exposure on the female reproductive tract.. Dev Biol.

[r11] Couse JF, Korach KS (1999). Estrogen receptor null mice: what have we learned and where will they lead us?. Endocr Rev.

[r12] Couse JF, Korach KS (2004). Estrogen receptor-α mediates the detrimental effects of neonatal diethylstilbestrol (DES) exposure in the murine reproductive tract.. Toxicology.

[r13] Couse JF, Yates MM, Walker VR, Korach KS (2003). Characterization of the hypothalamic-pituitary-gonadal axis in estrogen receptor (ER) null mice reveals hypergonadism and endocrine sex reversal in females lacking ERα but not ERβ.. Mol Endocrinol.

[r14] Diamanti-Kandarakis E, Bourguignon JP, Giudice LC, Hauser R, Prins GS, Soto AM (2009). Endocrine-disrupting chemicals: an Endocrine Society scientific statement.. Endocr Ref.

[r15] Dovey OM, Foster CT, Cowley SM (2010). Histone deacetylase 1 (HDAC1), but not HDAC2, controls embryonic stem cell differentiation.. Proc Natl Acad Sci USA.

[r16] Esteller M (2008). Epigenetics in cancer.. N Engl J Med.

[r17] Feinberg AP, Tycko B (2004). The history of cancer epigenetics.. Nat Rev Cancer.

[r18] Gabory A, Attig L, Junien C (2011). Developmental programming and epigenetics.. Am J Clin Nutr.

[r19] Greenwald P, Barlow JJ, Nasca PC, Burnett WS (1971). Vaginal cancer after maternal treatment with synthetic estrogens.. N Engl J Med.

[r20] Hall JM, McDonnell DP (2005). Coregulators in nuclear estrogen receptor action: from concept to therapeutic targeting.. Mol Interv.

[r21] Hayakawa T Nakayama J 2011 Physiological roles of class I HDAC complex and histone demethylase. J Biomed Biotechnol2011 129383; 10.1155/2011/129383 PMC2964911 21049000

[r22] Hendrich B, Tweedie S (2003). The methyl-CpG binding domain and the evolving role of DNA methylation in animals.. Trends Genet.

[r23] Henley DV Mueller S Korach KS 2009 The short-chain fatty acid methoxyacetic acid disrupts endogenous estrogen receptor-α–mediated signaling. Environ Health Perspect 117 1702 1706; 10.1289/ehp.0900800 20049119 PMC2801194

[r24] Herbst AL (2000). Behavior of estrogen-associated female genital tract cancer and its relation to neoplasia following intrauterine exposure to diethylstilbestrol (DES).. Gynecol Oncol.

[r25] Herbst AL, Ulfelder H, Poskanzer DC (1971). Adenocarcinoma of the vagina—association of maternal stilbestrol therapy with tumor appearance in young women.. N Engl J Med.

[r26] Hewitt SC, Li Y, Li L, Korach KS (2010). Estrogen-mediated regulation of Igf1 transcription and uterine growth involves direct binding of estrogen receptor α to estrogen-responsive elements.. J Biol Chem.

[r27] Higgins SJ, Burchell JM, Mainwaring WI (1976). Androgen-dependent synthesis of basic secretory proteins by the rat seminal vesicle.. Biochem J.

[r28] Higgins SJ, Colman A, Fuller FM, Jackson PJ (1981). Synthesis of androgen-dependent secretory proteins by rat seminal vesicles.. Mol Cell Endocrinol.

[r29] Institute for Laboratory Animal Research. (2011). Guide for the Care and Use of Laboratory Animals. 8th ed. Washington, DC:National Academies Press.. http://www.nap.edu/catalog.php?record_id=12910#.

[r30] Jaenisch R, Bird A (2003). Epigenetic regulation of gene expression: how the genome integrates intrinsic and environmental signals.. Nat Genet.

[r31] Jenuwein T, Allis CD (2001). Translating the histone code.. Science.

[r32] Jin VX, Leu YW, Liyanarachchi S, Sun H, Fan M, Nephew KP (2004). Identifying estrogen receptor α target genes using integrated computational genomics and chromatin immunoprecipitation microarray.. Nucleic Acids Res.

[r33] Jones PA, Baylin SB (2007). The epigenomics of cancer.. Cell.

[r34] Kandala JC, Kistler WS, Kistler MK (1985). Methylation of the rat seminal vesicle secretory protein IV gene. Extensive demethylation occurs in several male sex accessory glands.. J Biol Chem.

[r35] Li L (2009). GADEM: a genetic algorithm guided formation of spaced dyads coupled with an EM algorithm for motif discovery.. J Comput Biol.

[r36] Li S, Washburn KA, Moore R, Uno T, Teng C, Newbold RR (1997). Developmental exposure to diethylstilbestrol elicits demethylation of estrogen-responsive lactoferrin gene in mouse uterus.. Cancer Res.

[r37] Liu Y, Teng CT (1992). Estrogen response module of the mouse lactoferrin gene contains overlapping chicken ovalbumin upstream promoter transcription factor and estrogen receptor-binding elements.. Mol Endocrinol.

[r38] Liu Y, Yang N, Teng CT (1993). COUP-TF acts as a competitive repressor for estrogen receptor-mediated activation of the mouse lactoferrin gene.. Mol Cell Biol.

[r39] Luger K, Mader AW, Richmond RK, Sargent DF, Richmond TJ (1997). Crystal structure of the nucleosome core particle at 2.8 Å resolution.. Nature.

[r40] Marselos M, Tomatis L (1992a). Diethylstilboestrol: I, pharmacology, toxicology and carcinogenicity in humans.. Eur J Cancer.

[r41] Marselos M, Tomatis L (1992b). Diethylstilboestrol: II, pharmacology, toxicology and carcinogenicity in experimental animals.. Eur J Cancer.

[r42] McClain RM Keller D Casciano D Fu P MacDonald J Popp J 2001 Neonatal mouse model: review of methods and results. Toxicol Pathol(29 suppl 128 137 11695548 10.1080/019262301753178537

[r43] McLachlan JA (1977). Prenatal exposure to diethylstilbestrol in mice: toxicological studies.. J Toxicol Environ Health.

[r44] McLachlan JA, Dixon RL (1977). Toxicologic comparisons of experimental and clinical exposure to diethylstilbestrol during gestation.. Adv Sex Horm Res.

[r45] Moggs JG Ashby J Tinwell H Lim FL Moore DJ Kimber I 2004 The need to decide if all estrogens are intrinsically similar. Environ Health Perspect 112 1137 1142; 10.1289/ehp.7028 15289156 PMC1247471

[r46] Newbold RR, Bullock BC, McLachlan JA (1990). Uterine adenocarcinoma in mice following developmental treatment with estrogens: a model for hormonal carcinogenesis.. Cancer Res.

[r47] Newbold RR, Hanson RB, Jefferson WN (1997). Ontogeny of lactoferrin in the developing mouse uterus: a marker of early hormone response.. Biol Reprod.

[r48] Newbold RR, Pentecost BT, Yamashita S, Lum K, Miller JV, Nelson P (1989). Female gene expression in the seminal vesicle of mice after prenatal exposure to diethylstilbestrol.. Endocrinology.

[r49] Pentecost BT, Teng CT (1987). Lactotransferrin is the major estrogen inducible protein of mouse uterine secretions.. J Biol Chem.

[r50] Prins GS (1992). Neonatal estrogen exposure induces lobe-specific alterations in adult rat prostate androgen receptor expression.. Endocrinology.

[r51] Prins GS, Birch L, Couse JF, Choi I, Katzenellenbogen B, Korach KS (2001). Estrogen imprinting of the developing prostate gland is mediated through stromal estrogen receptor α: studies with αERKO and βERKO mice.. Cancer Res.

[r52] Prins GS, Bremner W (2004). Andrology in the 20th century: a commentary on our progress during the past 25 years.. J Androl.

[r53] Prins GS, Woodham C, Lepinske M, Birch L (1993). Effects of neonatal estrogen exposure on prostatic secretory genes and their correlation with androgen receptor expression in the separate prostate lobes of the adult rat.. Endocrinology.

[r54] Sato K, Fukata H, Kogo Y, Ohgane J, Shiota K, Mori C (2006). Neonatal exposure to diethylstilbestrol alters the expression of DNA methyltransferases and methylation of genomic DNA in the epididymis of mice.. Endocr J.

[r55] Sato K, Fukata H, Kogo Y, Ohgane J, Shiota K, Mori C (2009). Neonatal exposure to diethylstilbestrol alters expression of DNA methyltransferases and methylation of genomic DNA in the mouse uterus.. Endocr J.

[r56] Tang WY, Morey LM, Cheung YY, Birch L, Prins GS, Ho SM (2012). Neonatal exposure to estradiol/bisphenol A alters promoter methylation and expression of *Nsbp1* and *Hpcal1* genes and transcriptional programs of *Dnmt3a/b* and *Mbd2/4* in the rat prostate gland throughout life.. Endocrinology.

[r57] Teng CT (2002). Lactoferrin gene expression and regulation: an overview.. Biochem Cell Biol.

[r58] Turner T, Edery M, Mills KT, Bern HA (1989). Influence of neonatal diethylstilbestrol treatment on androgen and estrogen receptor levels in the mouse anterior prostate, ventral prostate and seminal vesicle.. J Steroid Biochem.

[r59] Wade PA (2001). Methyl CpG-binding proteins and transcriptional repression.. Bioessays.

[r60] Wade PA (2005). SWItching off methylated DNA.. Nat Genet.

[r61] Walker VR Jefferson WN Couse JF Korach KS 2012 Estrogen receptor-α mediates diethylstilbestrol-induced feminization of the seminal vesicle in male mice. Environ Health Perspect 120 560 565; 10.1289/ehp.1103678 22275727 PMC3339448

[r62] Wu SC, Zhang Y (2010). Active DNA demethylation: many roads lead to Rome.. Nat Rev Mol Cell Biol.

[r63] Yamamoto T, Matsuda T, Junicho A, Kishi H, Saatcioglu F, Muraguchi A (2000). Cross-talk between signal transducer and activator of transcription 3 and estrogen receptor signaling.. FEBS Lett.

