# Supplementary material for: Diethylstilbestrol (DES)-Stimulated Hormonal Toxicity is Mediated by ERα Alteration of Target Gene Methylation Patterns and Epigenetic Modifiers (DNMT3A, MBD2, and HDAC2) in the Mouse Seminal Vesicle
Source: Environ Health Perspect. 2013 Dec 6;122(3):262–8. doi: 10.1289/ehp.1307351 (PMC3948038; doi:10.1289/ehp.1307351)

Environmental Health Perspectives doi:10.1289/ehp.1307351

Supplemental Material

Diethylstilbestrol (DES)-Stimulated Hormonal Toxicity is Mediated by ERα Alteration of Target Gene Methylation Patterns and Epigenetic Modifiers (*DNMT3A*, *MBD2*, and *HDAC2*) in the Mouse Seminal Vesicle

Yin Li, Katherine J. Hamilton, Anne Y. Lai, Katherine A. Burns, Leping Li, Paul A. Wade, and

Kenneth S. Korach

Table of Contests

Supplemental Material, Table S1.. 2
Supplemental Material, Table S2.. 3
Supplemental Material, Method.. 4
Supplemental Material, Figure S1... 5
Supplemental Material, Figure S2... 6
Supplemental Material, Figure S3... 7
Supplemental Material, Figure S4... 8
Supplemental Material, Figure S5... 9
Supplemental Material, Figure S6... 10

Supplemental Material, Table S1. Real-time PCR primers.

**Gene symbol and GenBank accession number**	**Sequence (5'–3')**
Mouse *Svs4* (NM_009300)	F: GGAGCCATATCGCCAGAACA<br>R: TCCAGGGACTGCCTCTGAGA
Mouse *Ltf* (NM_008522)	F: CAGCAGGATGTGATAGCCACAA<br>R: CACTGATCACACTTGCGCTTCT
Mouse *Pgr* (NM_008829)	F: GATGAAGCATCTGGCTGTCACT<br>R: TTATGCTGAACTTCCATTGC
Mouse *Stat3* (NM_011486)	F: CCTCTATCAGCACAACCTTCG<br>R: CCACGATCCGGGCAATT
Mouse *Stat5a* (NM_ 001164062)	F: CCGTGGGATGCTATTGA<br>R: GAGTTGGGTGGCCTGAC
Mouse *DNMT1* (NM_001199431)	F: GGAAGGCTACCTGGCTAAAGTCAAG<br>R: ACTGAAAGGGTGTCACTGTCCGAC
Mouse *DNMT3A* (NM_001271753)	F: TGGAGAATGGCTGCTGTGTGAC<br>R: CACTCATCCCGTTTCCGTTTG
Mouse *DNMT3B* (NM_001003960)	F: AGTGACCAGTCCTCAGACACGAAG<br>R: ATCAGAGCCATTCCCATCATCTAC
Mouse *MeCP2* (NM_001081979)	F: CCTTGCTCTAGGGAGTAATC<br>R: TTGCACTCCATTGTCCAGCT
Mouse *MBD2* (NM_013594)	F: AACTTTCGACTGACCTTCCC<br>R: ACCTTTCCAGTAGACTGCAC
Mouse *MBD3* (NM_010773)	F: CCACACTGAAAGCAGATGAG<br>R: TCCAAACCCTGGCCTGTAAT
Mouse *HDAC1* (NM_008228)	F: AGGGACCCCTGCAATATCAA<br>R: CAACCTCCACCCTACAGAAT
Mouse *HDAC2* (NM_008229)	F: GGTCGTAGGAATGTTGCTGA<br>R: AGCCAATGTCCTCAAACAGG

Supplemental Material, Table S2. Bisulfite conversion sequencing primers.

Gene symbol and GenBank accession number	Sequence (5' – 3')
Mouse *Svs4* (NM_009300)	F: TTGTTAGAAAAGATTTTGATTGAAGG<br>R: TCCCTATACCATTTATCAATACAACA
Mouse *Ltf* (NM_008522)	F: GGTTAATTTTGGGTGGTATTT<br>R: AAACAACTACTCAAACCTAACCAC
Mouse *Pgr* (NM_008829)	F: TTTAGGAGATAGGGGAGGAGAAAAG<br>R: TAACTTCTACCCCAAAAAAAACTCC

Methods

Lactoferrin (LF) immunohistochemistry (IHC)

Mouse SVs were placed immediately into 10-13 ml of Bouin's solution after euthanasia. Tissues were rocked on an Adams Nutator (Clay Adams, Parsippany, NJ) in Bouin's solution for 24-30 h before the Bouin's solution was disposed of and 70% ethanol was added to the tissues. Tissues were processed and embedded in paraffin once the solution remained clear in 70% (w/v) ethanol for several hours. Embedded tissue was sectioned (7 μm) and placed onto charged-glass microscope slides for IHC staining. Anti-LF antibody (sc-14434) was purchased from Santa Cruz Biotechnology (Santa Cruz, CA). Slides were deparaffinized and immunostained according to the manufacturer's instructions with the AEC kit (Zymed Laboratory, South San Francisco, CA) and counterstained with hematoxylin (Sigma, St. Louis, MO).

Statistical analysis

Two-way ANOVA with Bonferroni post-test was performed using GraphPad Prism version 6.00 (***, $p < 0.001$, Supplemental Material, Figures S4A and S5).

Supplemental Material, Figure S1. Immunohistochemistry of LF in SVs of WT and αERKO 10-weeks old male mice. Mouse SVs were fixed as described in Supplemental Material, Methods. Slides were deparaffinized, immunostained with anti-LF-antibody, and counterstained with hematoxylin. A strong staining of LF was found in WT DES group samples. (Magnification, 100x)

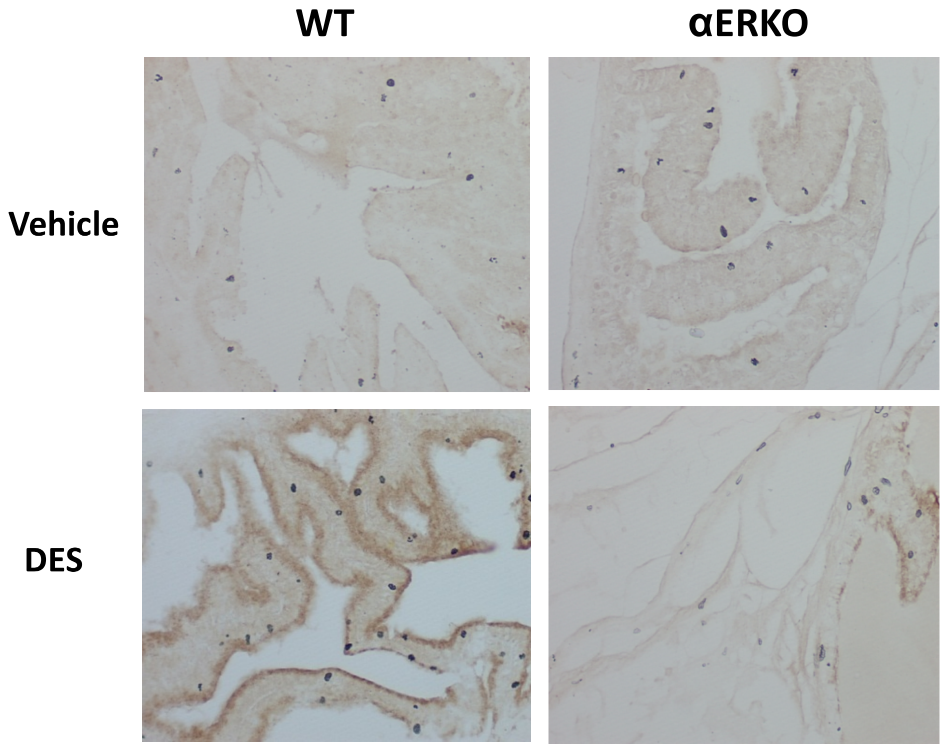

Supplemental Material, Figure S2. The 2 kb sequence surrounding the transcription start site (+/-1 kb) of the *Svs4* gene. The underlined sequence is the upstream 1 kb promoter sequence as the gene is transcribed from the minus strand. The black bold ACT is the transcription start site (TSS). Teal highlights represent the CpG positions tested in this study.

CpG positions are -160, -237, -306, and -367 bps upstream of TSS.

TATTTCCTGGAATTC is putative Stat5a/5b site at -132/-146 bp upstream of TSS.

The red sequence is a predicted Sp1 site (+118/+128 bp).

The blue sequence is a predicted ERE.

>mm10 *Svs4* chr2: 164277307-164279307

TCTGACAGTTTCTTCCGACTGCAAGAATTTTTCTGATGAGAGAAAAACAGGCTTTGCTCTCTAC
CTGTATATTTCCATATAGTCTGGGATTGTTATAAACCAAGACAAGGCAAACAACCTTTGCTTTG
ATTAGTAACCTATCTTGTGATGCAATTTGCCATTTTTGTTGTCTAGAAATTCCTTGATCTTTTGT
CTAGTCAGAAATAGGCTTAATTGTGAGCGCCTGACTCACCAGCAAAGCAGACACCACAACAG
GATCCTTCTACAACATGCCCCGAGCCCAGAACTGGTGCTGCTTATATAGGCCTAGGAGAGGCG
TGTCTCACACCTGGATTGGTTATGCATTCTACCTCATTTGCATGTCTCTCATCTGATTGGTTATT
CTCTCTCTCTCAGTACCTCACTGAGCCTCATTATCATACCTCATTTGCATGTCTCTCATCTGATT
GGTGATTCTCTCTCAGTATCTCATGGAGCCTCATTATCATGCCTAGGCCAGGCAGTGACTTGGT
GAAAAACTTTACTGCATATGTACACATTGGTTGTTTACCCAGACTTATGCGAGGTGGCCAGCG
GTAGCCAGCGCCACCTTGTAATGGCATATGTGGCTTCCCACACTTAATCTGTTTGGAAACTGCC
CTTGATACACTCTCCTTATAATGAACAATAAGAATCTTTAAGTTACATGACCTCCAACTGTTCC
CTTGCAGTCTACATCTCTGATTCTTGGTTTATCAATTCAGTGATACCCACACAAAAGCTATGGT
TACAGCTAGCAAAGACTAACTTCTCATCAGAAATTAGAGTCTCTAGAAAGTAACGCTCTTTAG
TTCCTCAGAGTACCCTTTAAAGAACCCTGTCATTGTTTCTTCT**CCCCCCCCCCC**ATCAACTCTT
CCTACTCACCCTTAGTTTTCTTCCCAATGGCTCCTGTCACCAGAAGGAGAAGCAGAGAAAAGA
GGAACAA**ACT**GGTAGAATTCATCTTGCCAGAAAAGATCTTGACTGAAGGCTAAGCTGAGTTCT
TAATTTATATATTTCTCAGGTGGGTGTCCCTGGAGTTATAAAAGGCACACCCCACCTTACTTAT
CTGTAAATTCTGGCTTCTTCCCCTGTGCTATGCAGGGGGTATTGTCAT**TATTTCCTGGAATTC**T
ATAAAAGCACATCGTATCAGAGACTTTCTTTTATACCTCCAACTTCCAGCCAATCTTTATCAAC
ACTTGAGATCATTTCACACAGTTTCCGGCATTTCTGAAAGACATTCAGTTGGGACTGAAGAAG
ACTGCCCCTTCATGGTACAAAGTGACTATCCCGTAATCAATATTTCCACAGCACAAGTGTATGT
CTCTTTCATATACATCCTATAACTTTGCGCTTATGATTCTCTCAAATAAACAAATATGTTGTACT
GACAAATGGCACAGGGATAGAAAGACACAGAATGAACTCAAGAAATCCAAATGAAAACCTCA
ATAGGGCAGCTAGAAGAACTTCAGGGGAAAGCCTCACCCACAGAAT**GGGTCATGTCGGAAT**
TGGAAGGTAGAGGATGGAGAACACATAAAGGCATGGAATCATTCGGGGAAGCTCAATACGAT
TTTTTTTTTTTTTGATGCATCAGTGGAACCACGGGAGAGTTTTGAGACACTGAGAAAAAGATGT
AACCTCTGAGTTGTGGAGCTAGAGGACAGATGAAAGAACAGCAGAGCAAAGGCATAGAGAAC
ATCTTCCATAAAAGCATAAAAAAGTCAAAACCCTAAAAAAAGAGATGTCCATCTTTGCATAAG
AGGCTCACTGAACACCCAATAGAAAAGACCAGAAAAAAACTTCTCTATGGTGTAGTTAAAATC
TTTAAAACAGATAATAAAGGGTATTTAAAACTGACAGAAATGTCAAGTTACTTATAAAGGCAG
GCTCACCGGAATAACAAATGATAACAATAACAATAACAGGAATCTGTAAAGCCAGGAGGGCC
AAGAAAGTTCCAAGTCCTGAAAGATCACAACTGTC

Supplemental Material, Figure S3. The upstream 1 kb sequence from the transcription start site (TSS) of the *Ltf* gene. The black bold **CAG** is TSS. Teal highlights represent the CpG positions tested in this study.

CpG positions are -449, -459, -470, -528, -542 bps upstream of TSS.

The red and blue sequences are predicted EREs.

AGGTCANNNTGACCC is a consensus ERE site.

ERE (-324, well-known)
AGGTCAAGGTAACCC
|||||| | ||||
AGGTCANNNTGACCC

Predicted additional EREs
AGACCAGGCTGGCCT
|| || || ||
AGGTCANNNTGACC

TGGGGACTGTGACTC
|| | ||||| |
AGGTCANNNTGACCC

Good half site
AGGTCACCCAGCACA
|||||| | |
AGGTCANNNTGACCC

The green sequences are Sp1 sites.

>mm10 *Ltf* chr9: 111018292-111020291

CTGGAACTCACTCTGT**AGACCAGGCTGGCCT**GGAACTCAGAAATCTGCCTGTCTCTGTCTCCCAAGTG
CTGGGATTAAAGGCATGCGCCACCACCGCCCGGCTCATAATATCATAATATGACTCTTAAGACATTCA
TTAAAAAGAAGTTCTCATTTCTATACCACACATGTACACACACACACACAATCTCTCTCTCTCACACAC
ACATACACACACACACACACACACACACACACAGACTCACACACACAAACACACAGACACAAAAATAGA
CACGGTCACAGATAGACATAGACACAGACAGACACACAGAGGAAGAGGAAAAAAAGATATGGAGGA
AGGAATGAAAGAAAAGGAGCAAAAGAAATCAAATGTCAGGGTCTCTTCCTCGCTAGCAAATGAAGGG
ACACAGGTCAACCCTGGGTGGCATTCCTTTCTGAGGTCCTAGGTTATTTTCGGGGGCTGTATGGCGGGT
TTCAAGGCAGTGTGGACCCCACAGGAACCCTGTGTGCAAGTCTAGGCTGACTCCGCTCTCCCTGCGGC
TGTCACCGGGCTGCTGTTGTGGCCAGGCCTGAGCAGCTGCCTCTTCTTTAGAATCCACCACTCTTTGTC
TAGCCAAGGAGGAAGGGGATTTGCTTGCTCCATGCAGCTTAAGTGTCAC**AGGTCAAGGTAACCC**ACA
AATATAGA**CCCCCTACCCC**ATGTCCCACCTCTAGAAAGTACTGGAAACAGAGAAAGGAGAAGACT**T**
GGGGACTGTGACTCTGATCCTGCAGAAGCTGGGTGGAGATTAAGGAAATCACTCGGTTTCCTGTACC
AGCGCCTGTGTAGGGGGTACTGGAGTCCCTGTTTCCTCCTTCTGGGCTCCAGGAAGCTGGCCTCTAAGA
ACTAGCACACCTGGTTGAGGGCAATGGGGCTGGAAGGCAGGCCTATTGGGCAA**TAGGGTGGGGCC**A
GCCCGGTG**AGGTCACCCAGCACA**GATAAAGGCCCCG**GGGAGAGGG**CAGAAGC**CAG**

Figure S4. Expression and DNA methylation of the *Pgr* gene in week 10 WT or αERKO SV. (A) Total RNA samples were extracted from SV tissues of three individual 10-week old WT or αERKO mice after neonatal exposure to vehicle or DES. mRNA levels were quantified by real time-PCR. Data shown represent mean fold change (± SE) relative to the WT SV vehicle samples. (B) DNA methylation status of the *Pgr* gene. The region containing twenty CpGs was amplified by PCR from bisulfite-treated genomic DNA, and then the PCR product was subcloned into the pCR-TOPO-XL vector. The sequencing analysis of bisulfite-covered DNA was performed using CpGviewer program. Each line of circles indicates an individual clone sequenced in the analysis after bisulfite treatment and PCR. Open circles indicate un-methylated CpGs. Black circles indicate methylated CpGs. Yellow circles indicate undetectable CpGs (unknown).

A

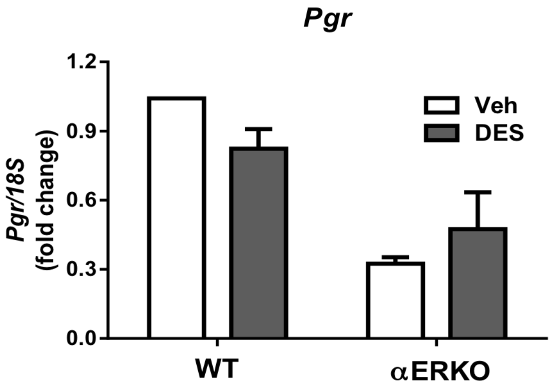

B

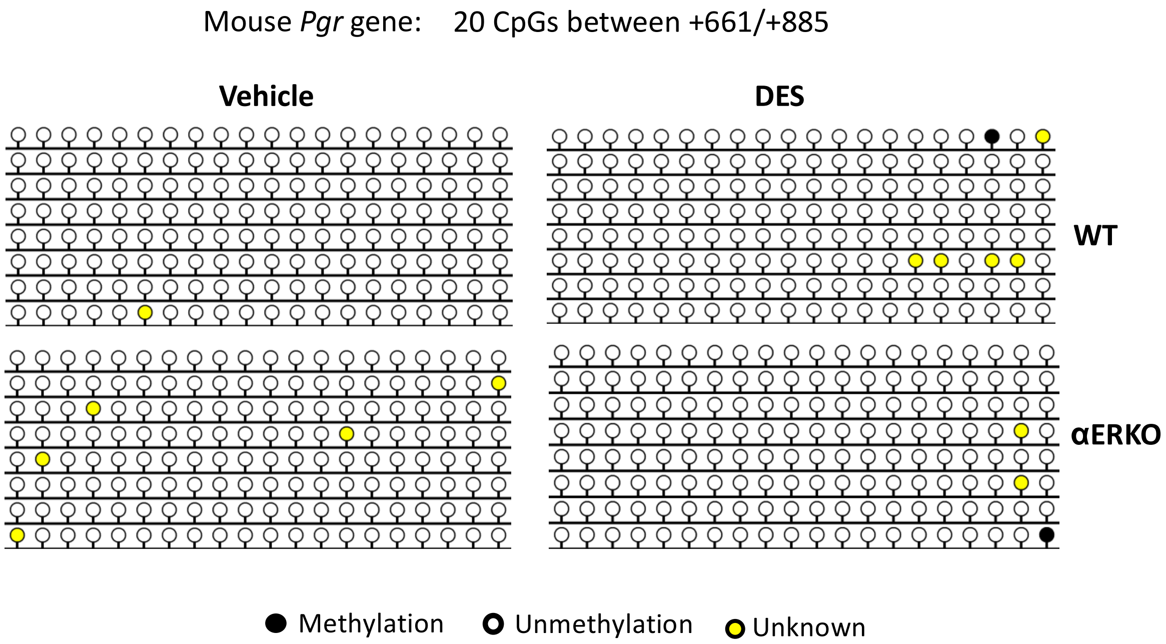

Supplemental Material, Figure S5. Changes in Signal transducer and activator of transcription (*Stat*) 3 or 5a after neonatal DES exposure in WT SV. Total RNA samples were extracted from SV tissues of individual 5- or 10-week old WT mice after neonatal exposure to vehicle or DES. The expression levels of *Stat3* and *Stat5a* were quantified by real time-PCR. Data shown represent mean fold change (± SE) relative to the WT SV vehicle samples at week 5, **p < 0.01.

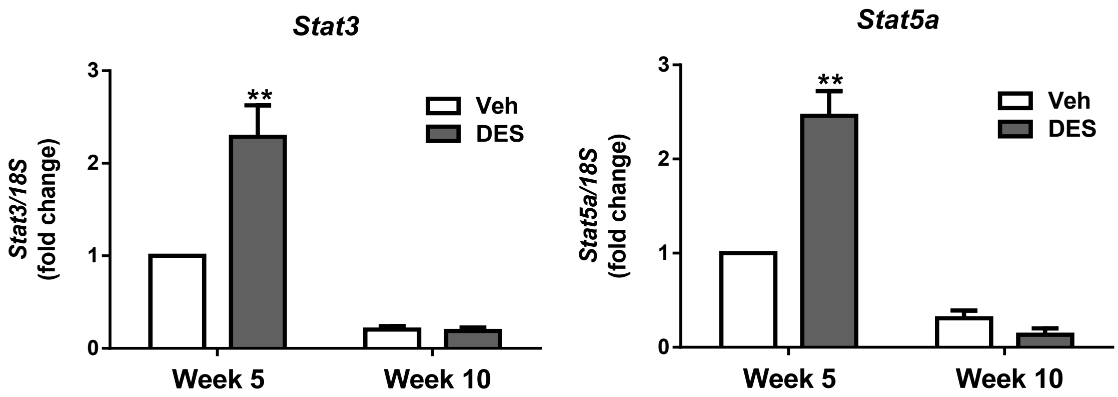

Supplemental Material, Figure S6. Working model. DES-induced toxicity is mediated partially by ERα; three epigenetic markers are involved in the epigenetic programming during neonatal DES exposure. For DNA methylation analysis, the 4 CpGs in the *Svs4* gene promoter changed from un-methylated to methylated and 2 CpGs in the *Ltf* gene promoter changed from methylated to un-methylated. These alterations of DNA methylation associate with the expression level of the genes.

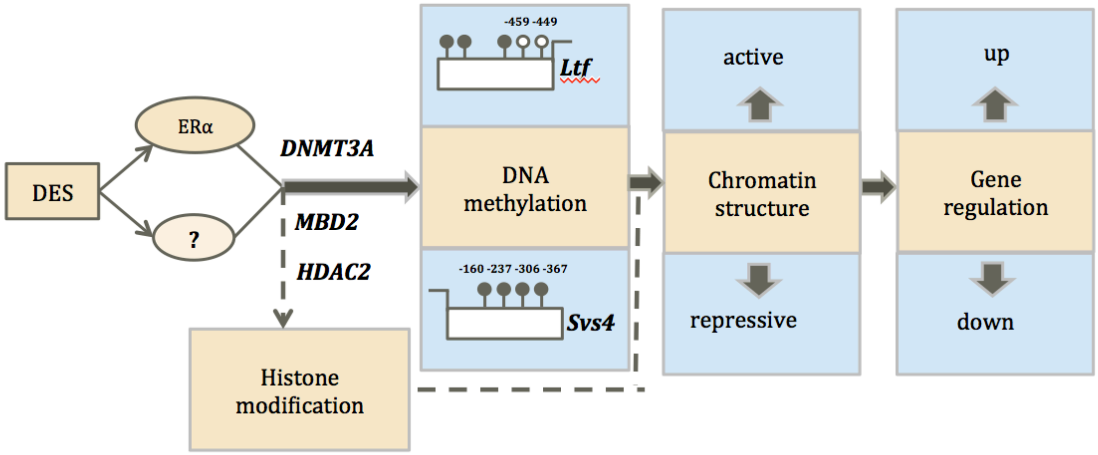

Supplement: (1.3 MB) PDF [file ehp.1307351.s001.pdf]
